# Effects of copper and zinc on proteoglycan metabolism in articular cartilage

**DOI:** 10.1155/S0962935196000154

**Published:** 1996-04

**Authors:** M. Pasqualicchio, R. Gasperini, G. P. Velo, M. E. Davies

**Affiliations:** 1 Strangeways Research Laboratory Worts' Causeway Cambridge CB1 4RN UK; 2 Institute of Pharmacology University of Verona Verona Italy

## Abstract

Co-Cultures of porcine articular cartilage and synovium or synovial conditioned medium were used as an *in vitro* model to mimic inflammatory events at the cartilage/synovial junction in degenerative joint disease. This model provides a useful tool to assess the anti-inflammatory and antiarthritic properties of pharmacological agents. In this study the effects of copper and zinc on (i) PG synthesis by cartilage and (ii) synovial-induced PG depletion have been investigated. Copper sulphate at a concentration of 0.01 mM did not stimulate PG synthesis significantly in cultured cartilage explants but completely abrogated the inhibitory effects of synovial tissue in co-culture experiments. This finding was supported by the histological demonstration of copper-dependent reversal of the PG depletion in cartilage exposed to synovial conditioned medium. Zinc sulphate at 0.01 mM had no effect on PG synthesis and was unable to protect cartilage against synovialinduced PG depletion. These results reveal possible mechanisms by which copper exerts its anti-inflammatory and anti-arthritic actions.


Mediators of Inflammation 5, 95–99 (1996)

### Effects of copper and zinc on proteoglycan metabolism in articular cartilage

M. Pasqualicchio,[1,2] R. Gasperini,[2] G. P. Velo[2] and M. E. Davies[1,CA]

[1]Strangeways Research Laboratory, Worts' Causeway, Cambridge CB1 4RN, UK. Fax: (+44) (0)1223 411609;
[2]Institute of Pharmacology, University of Verona, Verona, Italy

[CA]Corresponding Author

**Co-cultures of porcine articular cartilage and synovium or synovial conditioned medium were used as an *in vitro* model to mimic inflammatory events at the cartilage/synovial junction in degenerative joint disease. This model provides a useful tool to assess the anti-inflammatory and anti-arthritic properties of pharmacological agents. In this study the effects of copper and zinc on (i) PG synthesis by cartilage and (ii) synovial-induced PG depletion have been investigated. Copper sulphate at a concentration of 0.01 mM did not stimulate PG synthesis significantly in cultured cartilage explants but completely abrogated the inhibitory effects of synovial tissue in co-culture experiments. This finding was supported by the histological demonstration of copper-dependent reversal of the PG depletion in cartilage exposed to synovial conditioned medium. Zinc sulphate at 0.01 mM had no effect on PG synthesis and was unable to protect cartilage against synovial-induced PG depletion. These results reveal possible mechanisms by which copper exerts its anti-inflammatory and anti-arthritic actions.**

**Key words**: Anti-inflammatory agents, cartilage, copper, proteoglycan metabolism, synovium, zinc

### Introduction

Copper and zinc are essential trace minerals known to have important roles in immune[1–3] and inflammatory[4–6] processes. Adequate amounts of both minerals are required for normal maturation and function of the immune system, and deficiency, particularly of zinc, results in diminished immune responsiveness due to inhibition of T cell-mediated cytotoxicity[7] and antibody production.[8] It is also well established that dietary deficiency of copper and zinc correlates with enhanced acute inflammatory responses.[6,9,10]

Although the therapeutic anti-inflammatory properties of copper and zinc have been extensively studied, both in human disease and in animal models,[11–13] surprisingly little is known about their mechanisms of action. Even less information is available concerning their anti-arthritic action.[14–16] In order to gain some insight into anti-arthritic mechanisms we used a co-culture model of cartilage and synovial tissue to mimic events occurring at the cartilage/synovial pannus junction, the site of the initial pathological process of rheumatoid joint disease. As a result of an inflammatory reaction the synovial membrane becomes infiltrated by immunocompetent cells to form the pannus which invades the cartilage surface and underlying bone. Concomitant triggering of the cytokine network and production of degradative enzymes leads to depletion of the extracellular matrix macromolecules proteoglycan and collagen, and eventually to joint destruction. Our *in vitro* co-culture model provides a useful tool for studying the mechanism of action of pharmacological agents on the cellular and mediator interactions which play a role in these destructive pathological processes.

The aim of the present study was to investigate the ability of copper and zinc to abrogate the degradative effects of synovial tissue on cartilage matrix proteoglycan.

### Materials and Methods

*Tissue samples:* Macroscopically normal articular cartilage was obtained from the condylar ridge of metacarpo-phalangeal joints of pigs aged 6–9 months obtained from the local abattoir. Cartilage samples were collected into sterile Dulbecco's Modified Eagle's Medium (DMEM; Gibco) for subsequent culture experiments. Synovial villi and attached subsynovial tissue were dissected from the joint capsule lining of several metacarpo-phalangeal joints. The tissue was pooled and minced in preparation for co-culture as described previously.[17,18]

*Culture conditions:* Cultures were carried out under sterile conditions in serum-free DMEM,

© 1996 Rapid Science Publishers containing 0.58 mg/ml glutamine, 120 μg/ml benzyl penicillin, 200 μg/ml streptomycin, in a 5% $CO_2$ environment at 37°C. In the first experiment cartilage explants were cultured for 48 h in the presence or absence of a range of concentrations (0.01–2.0 mM) of copper sulphate or zinc sulphate in order to assess their effects on proteoglycan (PG) synthesis. From these data non-toxic concentrations were selected for further experiments in which cartilage explants were co-cultured in direct contact with minced synovium in the presence or absence of copper and zinc prior to measurement of PG synthesis.

In another group of experiments synovial tissue was pre-cultured for 48 h in the presence or absence of a range of copper sulphate concentrations (0.001–0.1 mM). The tissue-free synovial conditioned medium was then cultured with cartilage explants for a further 48 h, at which time the explants were removed, snap-frozen in OCT embedding medium (Miles Diagnostics) in liquid nitrogen and stored at −70°C prior to sectioning for toluidine blue histology. Five cartilage explants were used for each experimental group.

*Measurement of proteoglycan (PG) synthesis:* After culture in the presence of copper and zinc, or co-culture with synovial tissue, the explants were radiolabelled essentially as described previously[19] by placing fresh DMEM containing 20 μCi/ml (0.74 MBq/ml) of [$^{35}$S]-sulphate and incubating for 2 h at 37°C. The explants were then digested in papain (300 μg/ml) for 2 h at 65°C and the PG synthesis rate determined as the $^{35}SO_4^{2-}$ incorporation, and expressed as CPM/mg dry weight of cartilage.

*Toluidine blue histology:* Frozen sections 5–8 μm thick were cryostat cut at −25–35°C from cartilage explants exposed to synovial conditioned medium. The sections were placed on poly-L-lysine (Sigma) 8-well multitest slides (Flow Labs) and stained with toluidine blue for visual assessment of proteoglycan depletion.

*Statistics:* Proteoglycan synthesis measurements were performed in quintuplicate in every experiment. The dose–response experiments of copper and zinc on proteoglycan synthesis were done twice. The co-culture experiments were done three times with copper and twice with zinc, and the synovial conditioned medium experiment was done only once. Statistical evaluation of the data was done using Student's *t*-test.

### Results

*Effects of copper and zinc on PG synthesis by cartilage explants:* In two separate experiments copper sulphate at concentrations of 0.1 mM and below had little effect on PG synthesis in 48 h cartilage cultures. Since there was wide variation in basal PG synthesis between the two experiments the results were not combined. The results from a typical experiment are shown in Fig. 1(A). Higher concentrations of copper (0.25–2.0 mM) caused a dose-dependent decrease in PG synthesis.

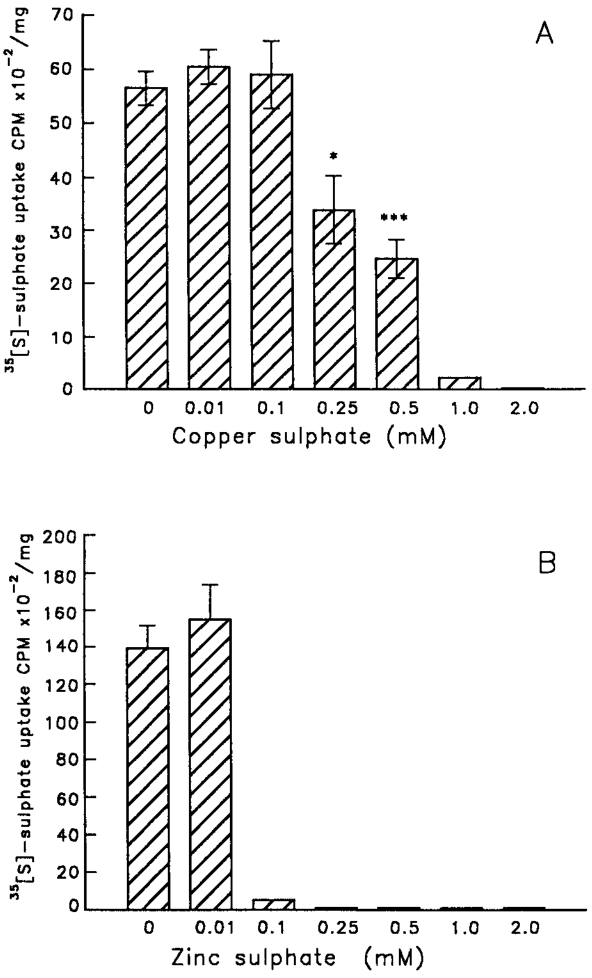

FIG. 1. Proteoglycan synthesis, expressed as CPM $^{35}SO_4$ uptake in articular cartilage cultured for 48 h alone and in the presence of increasing concentrations of copper sulphate (A) or zinc sulphate (B). (Means ± S.E.M. (n = 5); *$p < 0.05$; ***$p < 0.001$.)

Zinc sulphate at 0.01 mM caused a slight but non-significant increase in PG synthesis (Fig. 1(B)). Concentrations of zinc above 0.01 mM caused a dramatic and complete inhibition of PG synthesis.

From these results we selected a concentration of 0.01 mM for both copper and zinc sulphate in the co-culture experiments. Previous determination of toxic levels for cartilage and isolated chondrocytes[20] confirm that 0.01 mM is non-

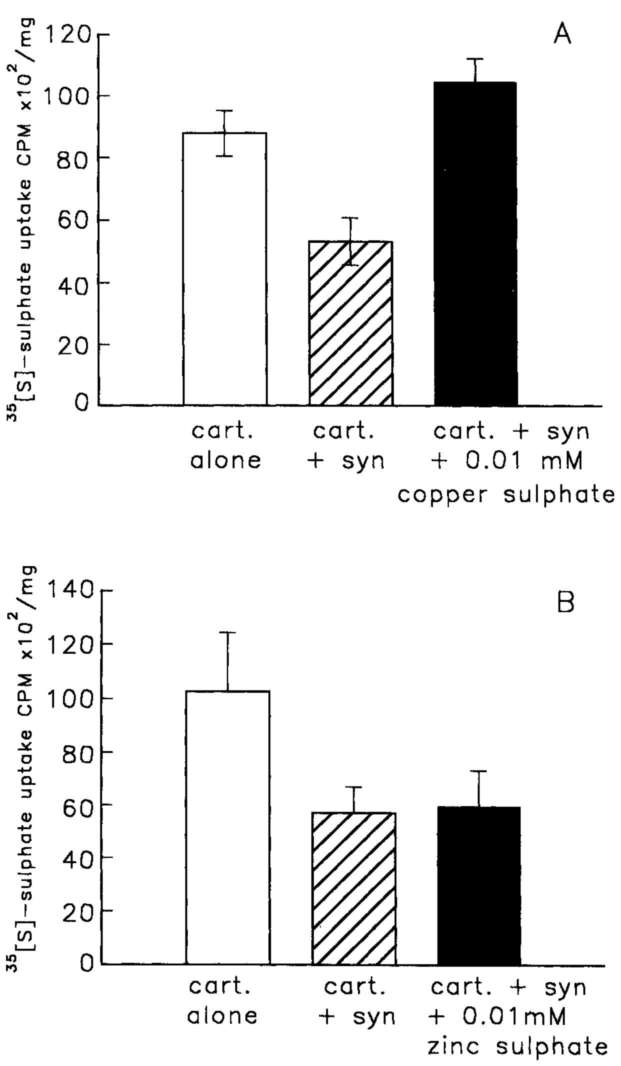

FIG. 2. Proteoglycan synthesis expressed as CPM $^{35}SO_4$ uptake in articular cartilage cultured for 48 h alone (□); co-cultured with synovium alone (▨) co-cultured with synovium in the presence of 0.01 mM copper sulphate (■) or 0.01 mM zinc sulphate (■). (Means ± S.E.M. ($n = 5$); $^{**}p < 0.01$.)

toxic and well within the range of concentrations used in other studies.[3,21,22]

*Effects of copper and zinc on PG synthesis by cartilage co-cultured with synovium:* Co-culture of cartilage with synovial tissue for 48 h resulted in marked reduction in PG synthesis. Figure 2(A) shows the results of a typical experiment in which synovial tissue caused a 40% reduction in PG synthesis by cartilage. The presence of copper sulphate at a concentration of 0.01 mM during the 48 h culture period abrogated the inhibitory effects of synovium ($p < 0.01$).

Zinc sulphate at a concentration of 0.01 mM was unable to reverse the inhibitory effects of synovial tissue on PG synthesis (Fig. 2(B)).

*Effect of conditioned medium from copper-treated synovium on PG content in cultured cartilage:* Toluidine blue staining of sulphated PG in cartilage sections allows good qualitative assessment of PG content in the extracellular matrix.[17] Figure 3 illustrates typical staining patterns seen in frozen sections prepared from the explants from the five different experimental groups. Figure 3(A) shows uniform metachromatic staining throughout the matrix, with slight depletion at the tip, in cartilage cultured alone for 48 h. Cartilage cultured in synovial conditioned medium shows characteristic extensive loss of metachromasia (Fig. 3(B)). Pre-treatment of synovium with 0.001 mM copper sulphate had little effect on the depletion of metachromasia (Fig. 3(C)) but higher concentrations increased the intensity of staining (0.01 mM, Fig. 3(D)) indicating markedly reduced loss of metachromasia induced by the copper-pretreatment conditioned medium (0.1 mM, Fig. 3(E)).

### Discussion

Experimental studies show that copper and zinc are involved in the expression and control of inflammation. How their metabolism changes in inflammation and how they can affect the development of inflammatory processes is unknown. In an attempt to gain insight into their possible anti-inflammatory and anti-arthritic mechanisms, we have investigated the effects of copper and zinc in an *in vitro* model of rheumatoid joint inflammation.

Our results presented here show that, co-culture of articular cartilage in direct contact with synovium, drastically reduced PG synthesis by chondrocytes (40%). When copper (0.01 mM) was added to the co-culture, the $^{35}SO_4$ incorporation into the glycosaminoglycans indicated a significant recovery of PG synthesis to reach the control values. On the contrary, zinc at the same concentration (0.01 mM) was unable to protect cartilage against synovial-induced PG depletion. The same inhibitory effect on PG synthesis has also been found by pre-culturing the synovium for 2 days alone and then adding the synovial conditioned medium (SCM) to the cartilage explants for 2 more days. In fact, cartilage cultured in SCM alone showed a pronounced loss of toluidine blue staining, reflecting depletion of matrix PG. This extensive reduction of metachromasia did not occur if the synovium had been pre-cultured in the presence of copper (0.01 mM) and if copper was still present in SCM added to cartilage, demonstrating a copper-dependent reversal of PG depletion in cartilage exposed to SCM.

From these results we can conclude that (i) the PG-degrading effect of synovium on cartilage

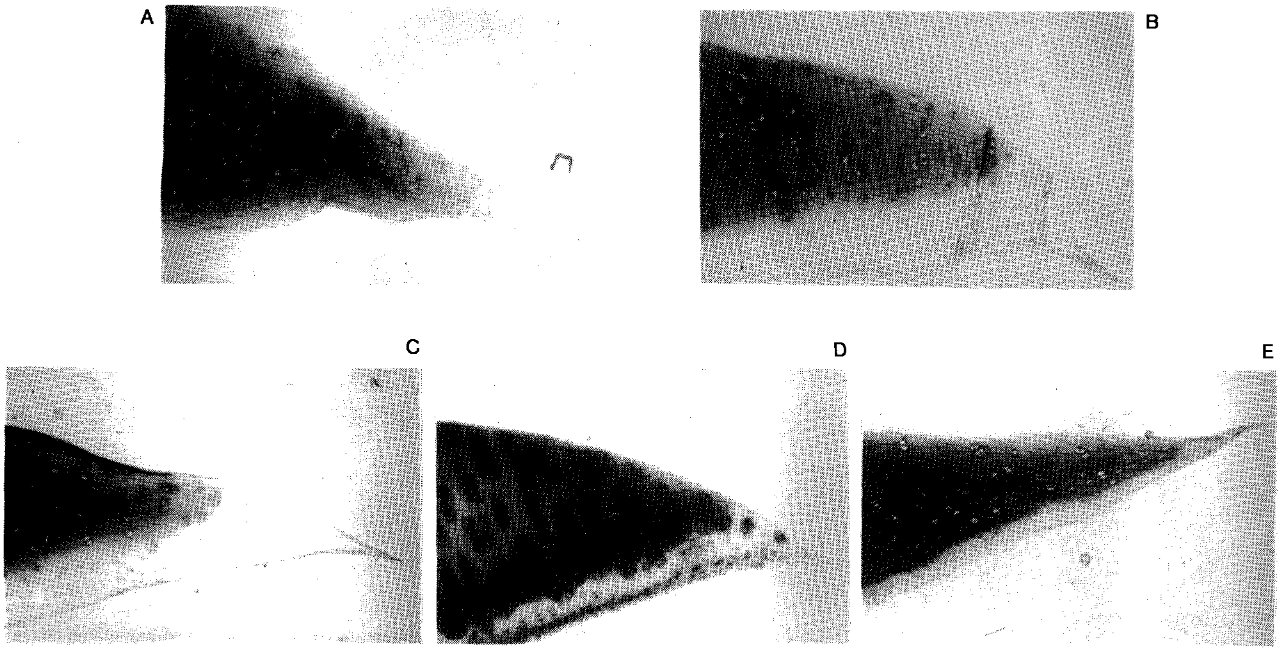

FIG. 3. Toluidine blue staining in frozen sections of articular cartilage cultured for 48 h alone (A), with 48 h synovial conditioned medium (B) or with conditioned medium from synovium pre-treated for 48 h in the presence of 0.001 mM (C), 0.01 mM (D) or 0.1 mM (E) copper sulphate.

does not necessarily require direct synovium-cartilage contact, but is likely attributable to mediators released from the synovium into the medium; (ii) copper is protective on cartilage matrix depletion either in co-culture or if the two tissues are not simultaneously present in the culture; and (iii) zinc is unable to reverse the PG depletion by synovium.

Because copper was always present in the medium, these results cannot distinguish between an inhibitory effect of copper on synovium or a stimulatory effect on PG synthesis in cartilage. However, we have also shown that copper added to the medium at a concentration of 0.01 mM did not increase PG synthesis in cartilage cultured alone, supporting indirectly an action of copper via synovium inhibition.

The original studies of Fell and Jubb[17] in 1977 demonstrated the ability of porcine synovial tissue to promote loss of PG from cartilage, and it is now known that interleukin 1 (IL-1) is one of the synovial mediators responsible for this degradative activity.[23,24] Interestingly, recent reports have shown that copper is able to inhibit IL-1 activity[25] and secretion,[21] findings which may explain our observations that copper protects cartilage from synovium-induced destruction. At present we have no direct evidence that copper inhibits IL-1 in our co-culture system.

An alternative mechanism by which copper may exert its protective action is via inhibition of the free radical, nitric oxide (NO). Increasing evidence implicates NO in the pathophysiology of arthritis,[26] suggesting a role for NO in both the inflammation and the chondrodepletion that occurs in this disease. Recent data obtained by Taskiran and co-workers[27] demonstrate that endogenously synthesized NO acts as the mediator of IL-1-dependent PG synthesis suppression. Analyses of human synovial fluids suggest that NO is synthesized locally within joints[28] and that synoviocytes can potentially serve as a source of intra-articular NO.[29]

In support of this we have preliminary data showing NO production by normal pig synovium (data not shown), and furthermore we have found a 50% reduction of NO content in SCM when synovium was cultured in the presence of non-toxic concentrations of copper (0.001–0.01 mM). The same SCM also abrogated the degradative effects of synovial tissue on cartilage, suggesting a connection between copper, NO reduction and cartilage protection. In the light of these results and because Scuderi[21] has recently shown that copper may play an important role in the homeostasis of cytokines such as IL-1,[21] we could hypothesize an action of copper on PG metabolism as indicated in Fig. 4.

The question as to whether copper affects NO production directly or via modulation of IL-1 synthesis and/or secretion is under investigation. In conclusion copper, in contrast to zinc, seems to be able to protect cartilage from the destructive effects of mediators released by synovium.

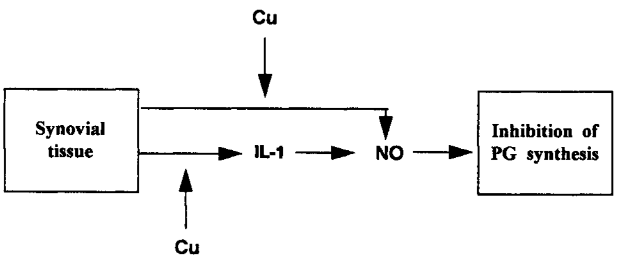

FIG. 4. A possible mechanism of action of copper on PG metabolism.

Because loss of articular cartilage is the major pathological lesion common to all arthritides, copper chondroprotection may explain the therapeutic efficacy of this metal in maintaining cartilage integrity during the inflammation process.

### References

1. Sullivan JL, Ochs HD. Copper deficiency and the immune system. *Lancet* 1978; **2**: 686.
2. Niederman CN, Blodgett D, Eversole D, Schurig GG, Thatcher CD. Effect of copper and iron on neutrophil function and humoral immunity of gestating beef cattle. *J Am Vet Assoc* 1994; **204**: 1796–1800.
3. Miller GG, Strittmatter WJ. Identification of human T cells that require zinc for growth. *Scand J Immunol* 1992; **36**: 269–277.
4. Milanino R, Marrella M, Gasperini R, Pasqualicchio M, Velo G. Copper and zinc body levels in inflammation: an overview of the data obtained from animal and human studies. *Agents Actions* 1993; **39**: 195–209.
5. Schuschke DA, Saari JT, Miller FN. The role of the mast cell in acute inflammatory responses of copper-deficient rats. *Agents Actions* 1994; **42**: 19–24.
6. Milanino R, Rainsford KD, Velo GP. *Copper and Zinc in Inflammation.* Kluwer, Dordrecht: Kluwer Academic Publishers, 1989.
7. Fernandez G, Nair M, Onoe K, Tanaka T, Floyd R, Good RA. Impairment of cell-mediated immunity functions by dietary zinc deficiency in mice. *Proc Natl Acad Sci USA* 1979; **76**: 457–461.
8. Weiyi Y. Einfluss von Zincmangel auf die Immunoglobulinproduktion beim Rind. Vet. Ph.D. Thesis, 1992 Tierärztliche Hochschule, Hannover, Germany.
9. Milanino R, Conforti A, Franco L, Marrella M, Velo GP. Copper and inflammation—a possible rationale for the pharmacological manipulation of inflammatory disorders. *Agents Actions* 1985; **16**: 506–513.
10. Denko CW, Petricevic M, Whitehouse MW. Inflammation in relation to dietary intake of zinc and copper. *Int J Tiss React* 1981; **3**: 73–76.
11. Alberghina M, Lupo G, La Spina G, Mangiameli A, Gulisano M, Sciotto D, Rizzarelli E. Cytoprotective effect of copper (II) complexes against ethanol-induced damage to rat gastric mucosa. *J Inorg Biochem* 1992; **45**: 245–259.
12. Jiménez-Hernandez RM, Frechilla D, Lasheras B, Gutiérrez-Rios MT, Parrondo E, Craciunescu G, Cenarruzabeita E. Inhibition of inflammation and gastric damage in rats by copper (II) complexes. *Arzneim Forsch/Drug Res* 1995; **45**: 277–281.
13. Nagai H, Kitagaki K, Kuwabara K, Koda A. Anti-inflammatory properties of zinc protoporphyrin disodium (Zn-PP-2Na). *Agents Actions* 1992; **37**: 273–283.
14. Whitehouse MW, Rainsford KD, Taylor RM, Vernon-Roberts B. Zinc monoglycerolate: a slow-release source of zinc with anti-arthritic activity in rats. *Agents Actions* 1990; **31**: 47–58.
15. Sorenson JRJ. Copper complexes offer a physiological approach to treatment of chronic diseases. In: Ellis GP, West GB, eds. *Progress in Medicinal Chemistry.* Vol. 26. Amsterdam: Elsevier, 1989; 437–568.
16. Milanino R, Frigo A, Bambara LM, Marrella M, Moretti U, Pasqualicchio M, Biasi D, Gasperini R, Mainenti L, Velo GP. Copper and zinc status in rheumatoid arthritis: studies on plasma, erythrocytes, and urine, and relationship with disease activity markers and pharmacological treatment. *Clin Exp Rheumatol* (in press).
17. Fell HB, Jubb RW. The effect of synovial tissue on the breakdown of articular cartilage in organ culture. *Arthritis Rheum* 1977; **20**: 1359–1371.
18. Davies ME, Horner A, Dingle JT. Immunorecognition of chondrocytes in articular cartilage activated by synovial interleukin 1. *Conn Tiss Res* 1991; **25**: 243–249.
19. Tyler JA. Chondrocyte-mediated depletion of articular cartilage proteoglycans *in vitro. Biochem J* 1985; **225**: 493–507.
20. Pasqualicchio M, Davies ME, Velo GP. Effects of copper and zinc on chondrocyte-mononuclear cell adhesion via ICAM-1/CD18 interactions. *Inflammopharmacol* 1995; **3**: 35–48.
21. Scuderi P. Differential effects of copper and zinc on human peripheral blood monocyte cytokine secretion. *Cell Immunol* 1990; **126**: 391–405.
22. Steinbach OM, Wolterbeek HTh. Effects of copper on rat hepatoma HTC cells and primary cultured rat hepatocytes. *J Inorg Biochem* 1994; **53**: 27–48.
23. Saklatvala J, Pilsworth LMC, Sarsfield SJ, Gavrilovic J, Heath JK. Pig catabolin is a form of IL-1. *Biochem J* 1984; **224**: 461–466.
24. Shingu M, Miyauchi S, Nagai S, Yasutake C, Horie K. The role of IL-4 in IL-1-dependent cartilage matrix degradation. *Br J Rheumatol* 1995; **34**: 101–106.
25. Vinci C, Caltabiano V, Santoro AM, *et al.* Copper addition prevents the inhibitory effects of interleukin 1β on rat pancreatic islets. *Diabetologica* 1995; **38**: 39–45.
26. Stefanovic-Racic M, Stadler J, Evans CH. Nitric oxide and arthritis. *Arthritis Rheum* 1995; **36**: 1036–1044.
27. Taskiran D, Stefanovic-Racic M, Georgescu H, Evans C. Nitric oxide mediates suppression of cartilage proteoglycan synthesis by IL-1. *Biochem Biophys Res Comm* 1994; **200**: 142–148.
28. Farrell AJ, Blake DR, Palmer RMJ, Moncada S. Increased concentration of nitrate in synovial fluid and serum samples suggest increased nitric oxide synthesis in rheumatic diseases. *Ann Rheum Dis* 1992; **51**: 1219–1222.
29. Stefanovic-Racic M, Stadler J, Georgescu HI, Evans CH. Nitric oxide synthesis and its regulation by rabbit synoviocytes. *J Rheumatol* 1994; **21**: 1892–1898.

ACKNOWLEDGEMENTS. M.P. gratefully acknowledges financial assistance from Unilever Research, Port Sunlight Laboratory, UK and Glaxo SpA, Verona Italy. M.E.D. thanks the Isaac Newton Trust and the Sybil Eastwood Trust for support. We are grateful to Laura Cuzzolin for performing the NO measurements.

**Received 19 October 1995; accepted in revised form 15 November 1996**
